# Supplementary material for: End Sequence Analysis Toolkit (ESAT) expands the extractable information from single-cell RNA-seq data
Source: Genome Res. 2016 Oct;26(10):1397–410. doi: 10.1101/gr.207902.116 (PMC5052061; doi:10.1101/gr.207902.116)
Supplement: Supplemental Material [file supp_gr.207902.116_Supp_Materials.tar.gz › ESAT-esat_v0.1/doc/html/classbroad_1_1pda_1_1samtools_1_1_paired_end_read_iterator.html]

ESAT: broad.pda.samtools.PairedEndReadIterator Class Reference


ESAT


- Main Page
- Related Pages
- Packages
- Classes
- Files

- Class List
- Class Index
- Class Hierarchy
- Class Members

All Classes Namespaces Files Functions Variables Enumerator Pages

- broad
- pda
- samtools
- PairedEndReadIterator

Public Member Functions |
List of all members

broad.pda.samtools.PairedEndReadIterator Class Reference

Inheritance diagram for broad.pda.samtools.PairedEndReadIterator:

[legend]

Collaboration diagram for broad.pda.samtools.PairedEndReadIterator:

[legend]

|  |  |
| --- | --- |
| Public Member Functions | |
|  | PairedEndReadIterator (SAMFileReader reader) |
|  | |
| boolean | hasNext () |
|  | |
| Pair< SAMRecord > | next () |
|  | |
| void | close () |
|  | |
| void | remove () |
|  | |
| int | getSingleReadsSkipped () |
|  | |

## Detailed Description

Author
:   engreitz This class iterators over a standard format SAM or BAM file, but gives an iterator over paired reads with the same name. Does not return reads that do not have a mate.

## Constructor & Destructor Documentation

|  |  |  |  |  |  |
| --- | --- | --- | --- | --- | --- |
| broad.pda.samtools.PairedEndReadIterator.PairedEndReadIterator | ( | SAMFileReader | *reader* | ) |  |

## Member Function Documentation

|  |  |  |  |  |
| --- | --- | --- | --- | --- |
| void broad.pda.samtools.PairedEndReadIterator.close | ( |  | ) |  |

|  |  |  |  |  |
| --- | --- | --- | --- | --- |
| int broad.pda.samtools.PairedEndReadIterator.getSingleReadsSkipped | ( |  | ) |  |

|  |  |  |  |  |
| --- | --- | --- | --- | --- |
| boolean broad.pda.samtools.PairedEndReadIterator.hasNext | ( |  | ) |  |

|  |  |  |  |  |
| --- | --- | --- | --- | --- |
| Pair<SAMRecord> broad.pda.samtools.PairedEndReadIterator.next | ( |  | ) |  |

|  |  |  |  |  |
| --- | --- | --- | --- | --- |
| void broad.pda.samtools.PairedEndReadIterator.remove | ( |  | ) |  |

---

The documentation for this class was generated from the following file:

- java/broad/pda/samtools/PairedEndReadIterator.java


---

Generated on Fri Aug 15 2014 15:11:01 for ESAT by  

 1.8.7
